# What settings have been linked to SARS-CoV-2 transmission clusters?

**DOI:** 10.12688/wellcomeopenres.15889.2

**Published:** 2020-06-05

**Authors:** Quentin J. Leclerc, Naomi M. Fuller, Lisa E. Knight, CMMID COVID-19 Working Group, Sebastian Funk, Gwenan M. Knight

**Affiliations:** 1 Department of Infectious Disease Epidemiology, Faculty of Epidemiology and Population Health, London School of Hygiene & Tropical Medicine, London, UK; 2 Centre for Mathematical Modelling of Infectious Diseases, London School of Hygiene & Tropical Medicine, London, UK; 3 GP registrar, Brecon Surgery, Gwent Deanery, UK

**Keywords:** SARS-CoV-2, COVID-19, coronavirus, cluster, transmission, settings, lockdown

## Abstract

**Background**: Concern about the health impact of novel coronavirus SARS-CoV-2 has resulted in widespread enforced reductions in people’s movement (“lockdowns”). However, there are increasing concerns about the severe economic and wider societal consequences of these measures. Some countries have begun to lift some of the rules on physical distancing in a stepwise manner, with differences in what these “exit strategies” entail and their timeframes. The aim of this work was to inform such exit strategies by exploring the types of indoor and outdoor settings where transmission of SARS-CoV-2 has been reported to occur and result in clusters of cases. Identifying potential settings that result in transmission clusters allows these to be kept under close surveillance and/or to remain closed as part of strategies that aim to avoid a resurgence in transmission following the lifting of lockdown measures.

**Methods**: We performed a systematic review of available literature and media reports to find settings reported in peer reviewed articles and media with these characteristics. These sources are curated and made available in an editable online database.

**Results**: We found many examples of SARS-CoV-2 clusters linked to a wide range of mostly indoor settings. Few reports came from schools, many from households, and an increasing number were reported in hospitals and elderly care settings across Europe.

**Conclusions:** We identified possible places that are linked to clusters of COVID-19 cases and could be closely monitored and/or remain closed in the first instance following the progressive removal of lockdown restrictions. However, in part due to the limits in surveillance capacities in many settings, the gathering of information such as cluster sizes and attack rates is limited in several ways: inherent recall bias, biased media reporting and missing data.

## Introduction

The novel coronavirus SARS-CoV-2, responsible for coronavirus disease 2019 (COVID-19), was first identified in Wuhan, China at the end of 2019, and has since spread around the world ( European Centre for Disease Prevention and Control, 2020). The capacity of the virus for human-to-human transmission, coupled with the lack of immunity in the population due to the novelty of SARS-CoV-2, has led to the implementation of severe reductions in people’s movements in an effort to reduce disease impact. These strong measures are broadly described as “lockdowns”. Due to the highly restrictive nature of lockdowns, and their impact on people’s health, wellbeing and finances, it is likely that such interventions cannot be sustained for prolonged periods of time, and will have to be lifted, at least to some extent, before an effective vaccine becomes available.

To successfully remove these lockdown restrictions while avoiding a resurgence in SARS-CoV-2 transmission, we must better understand in which types of settings the virus is most likely to be transmitted. Determining particular places that are linked to clusters of cases could reveal settings that are responsible for amplifying the heterogeneity in transmission that has been reported: potentially 80% of transmission is being caused by only 10% of infected individuals ( Endo *et al.*, 2020). Notably, the difference in transmission risk between households and larger communal settings is unclear, as is the difference between indoor and outdoor transmission.

Quantifying these differences in transmission can be further facilitated by the fact that, in many countries now under lockdown, intensive contact tracing of imported cases was performed in the early stages of the epidemic, resulting in the detection of clusters of cases. This data, on the first detected clusters in a country, can give knowledge of the types of settings facilitating transmission before intensive social and physical distancing took place.

The aim of our work is therefore to gather information on reported clusters of COVID-19 cases to determine types of settings in which SARS-CoV-2 transmission occurred. This could inform post-lockdown strategies by identifying places which should be kept under close surveillance and/or should still remain closed to avoid a resurgence in transmission.

## Methods

### Outline

We searched for scientific literature and media articles detailing clusters of SARS-CoV-2 transmission (details below) and extracted data into a Google Sheets file (accessible at https://bit.ly/3ar39ky; archived as *Underlying data* ( Leclerc *et al.*, 2020)). We defined “settings” as sites where transmission was recorded resulting in a cluster of cases. We restricted our definition of “cluster” to the first-generation cases that acquired the infection due to transmission in a single specific setting at a specific time. For example, if a person was infected on a cruise ship, and later infected additional people after disembarking, we would not consider that the latter were part of that “cruise ship cluster”, since they were not infected on the ship. We recorded the country and further details about the type of setting, the numbers of primary and secondary cases in the cluster, cluster sizes, and attack rates. We defined a case as a person reported to be infected with the SARS-CoV-2 virus, regardless of symptoms.

### Search strategy

References were found in four ways. Firstly, we performed a systematic literature review for COVID-19 clusters in PubMed on the 30th March 2020 (search term below). A total of 67 papers were found. Two reviewers (GMK and QJL) performed data extraction into the online database. We chose to only search this database and use peer reviewed articles as a quality threshold. We included data from English abstracts (where possible), but otherwise excluded non-English publications.

PubMed search: ("COVID-19"[All Fields] OR "COVID-2019"[All Fields] OR "severe acute respiratory syndrome coronavirus 2"[Supplementary Concept] OR "severe acute respiratory syndrome coronavirus 2"[All Fields] OR "2019-nCoV"[All Fields] OR "SARS-CoV-2"[All Fields] OR "2019nCoV"[All Fields] OR (("Wuhan"[All Fields] AND ("coronavirus"[MeSH Terms] OR "coronavirus"[All Fields])) AND (2019/12[PDAT] OR 2020[PDAT]))) AND cluster [All Fields]

Secondly, we used the online Google search engine to find media articles detailing settings of SARS-CoV-2 transmission in general. We searched for combinations of either “COVID”, “COVID-19”, "COVID-2019","severe acute respiratory syndrome coronavirus 2", "2019-nCoV”, "SARS-CoV-2”, "2019nCoV" or "coronavirus”, and the words “transmission cluster” (e.g. “COVID transmission cluster” or “SARS-CoV-2 transmission cluster”). We only included online articles in English. From the collated list of settings, we then performed a further search for transmission in each of these settings (week beginning 6th April 2020).

Thirdly, we investigated whether information on the settings in which the first 100 “transmission events” in countries with current COVID-19 outbreaks existed by searching for publicly available data sources. As substantial investigation of cases often occurs early in an outbreak, any clusters linked to the first ~100 cases in countries outside China could give information on the transmission of SARS-CoV-2 in the absence of any social distancing measures.

Finally, following the original publication of this article on 01/05/2020, we included a “Suggested updates” tab in our publicly available database ( https://bit.ly/3ar39ky). This allows other individuals to suggest new clusters we should include in our analysis. We review these suggestions regularly, and add those with sufficient detail to our “Latest updated results” tab. In this revised version, we have updated our analysis to include suggestions we reviewed up to 26/05/2020.

### Cluster characteristics and setting definition

With the above data, we then aimed to estimate both the final (proportion of people in that setting who became infected) and secondary (proportion of contacts of one case who became infected) attack rates in each setting. These were previously identified as key metrics, particularly within households, to estimate whether transmission is driven by a relatively small number of high-risk contacts ( Liu *et al.*, 2020).

We defined a setting when several reports mentioned clusters linked to spaces with certain characteristics. For example, “Religious” includes churches and mosques, while “Public” here means public communal shared spaces such as markets or welfare centres. Where settings were a mixture of indoor and outdoor spaces, we used a mixed indoor/outdoor classification.

## Results

We found evidence of SARS-CoV-2 transmission clusters for 201 events, which we classified into 22 types of settings ( Table 1 and Table 2). All the studies with relevant data are compiled in an online database (accessible at https://bit.ly/3ar39ky; see also *Underlying data* ( Leclerc *et al.*, 2020)). Many of the published reports with setting specific data came from China (47/201) and Singapore (51/201).

**Table 1.** Summary of gathered reported events as of 20th April 2020. Where only one study for this setting is reported, the minimum, maximum and median number of secondary cases in the cluster and/or total cluster size correspond to this single reported number (if given). Total cluster size accounts for all primary and secondary cases in the cluster. For references see the online database, accessible at https://bit.ly/3ar39ky.

Setting type	Number of reported events	Secondary cases			Total cluster size			Total number of cases across all clusters	Countries	Indoor / outdoor
		Min	Median	Max	Min	Median	Max			
Bar	12	2	9	16	3	13	80	319	Germany, Austria, Italy, Singapore, Japan, USA, Australia, New Zealand, Brazil	Indoor / outdoor
Building site	4	/	/	/	5	20.5	49	95	Singapore	Outdoor
Conference	5	/	/	/	3	10	89	148	Canada, Singapore, Japan, USA, New Zealand	Indoor / outdoor
Elderly care	17	/	/	/	5	19	167	638	UK, Canada, Scotland, France, Germany, Italy, USA, Japan, New Zealand, Luxembourg	Indoor
Food processing plant	9	2	2	2	3	84	518	1207	USA, Germany, Canada, Netherlands	Indoor
Funeral	1	3	3	3	4	4	4	4	USA	Indoor / outdoor
Hospital	9	1	3	14	2	10	118	224	China, Singapore, Italy, Taiwan, South Korea, Japan	Indoor
Hotel	2	/	/	/	3	5	7	10	Singapore	Indoor
Household	36	1	3	11	2	4	12	168	China, Italy, Vietnam, Taiwan, South Korea, Hong Kong, France	Indoor
Meal	17	1	3	10	2	5	47	134	Singapore, USA, Vietnam, China, South Korea, Japan	Indoor
Prison	4	351	351	351	66	226	353	871	USA, Ethiopia	Indoor
Public	4	/	/	/	10	10	27	57	China, Japan	Indoor / outdoor
Religious	15	1	18	52	2	23	130	570	USA, Singapore, South Korea, US, China, India, Netherlands, Germany	Indoor / outdoor
School	8	1	1	131	2	22	133	349	Singapore, France, USA, New Zealand, Australia, Sweden	Indoor / outdoor
Ship	5	619	619	619	78	662	1156	3597	Grand Princess, Diamond Princess, Ruby Princess, USS Theodore Roosevelt, Charles de Gaulle aircraft carrier	Indoor
Shipyard	1	/	/	/	22	22	22	22	Singapore	Indoor / outdoor
Shopping	9	5	10	19	7	20	163	361	China, Singapore, Peru, Mexico	Indoor / outdoor
Sport	6	1	1	1	2	7.5	65	95	South Korea, Singapore, Italy, Japan	Indoor / outdoor
Transport	1	1	1	1	3	3	3	3	China	Indoor
Wedding	3	/	/	/	13	43	98	154	Australia, New Zealand	Indoor / outdoor
Work	12	6	7	11	4	8.5	97	198	China, Singapore, South Korea, Germany	Indoor
Worker dormitories	21	/	/	/	3	24	797	1702	Singapore	Indoor

**Table 2.** Definitions used for each of our transmission setting types. The definitions describe in what environment transmission was deemed to occur.

Transmission setting	Definition
Bar	Indoor space such as a bar, club, pub, small live music venues etc.
Building site	Outdoor space where construction work takes place.
Conference	Indoor professional event with many people interacting and meeting, shaking hands, eating together, team activities, etc.
Elderly Care	Care homes for the elderly; includes staff and residents. Transmission can occur between staff and residents but also from visitors.
Food processing plant	Any establishment that processes food for human consumption, such as a meat or vegetable packing plant.
Funeral	Indoor or outdoor burial ceremony; includes close contact with others such as hugging, shaking hands, eating together, singing, praying, etc.
Hospital	Any transmission that occurs within a hospital between patients and/or staff, in a COVID19 ward or not.
Hotel	Any transmission that occurs within the hotel e.g. hotel rooms, shared spaces, reception desk, etc.
Household	Transmission between individuals in a shared living space
Meal	When people eat together. Meals included took place in restaurants, hotels, cafes, home, etc. Transmission occurs over a meal by speaking, sharing foods, touching the same surfaces, etc.
Prison	Any transmission that occurs within a prison between prisoners and/or staff.
Public	Where transmission occurs on public property and does not fall into any of the other settings e.g. park, welfare centre, foodbank, etc.
Religious	Transmission occurs at a religious event such as at mass, services, prayer time, choir practice, etc.
School	Childcare or learning environments (schools, nurseries, kindergartens etc). Includes staff and children.
Ship	Any ship at sea. Includes crew and/or passengers onboard.
Shipyard	Large indoor or outdoor space where ships are made or repaired. Includes those working on the ship as well as customers
Shopping	A shop or shopping centre. Includes customers and those working in the shop.
Sport	Participation in a sporting activity indoor or outdoor e.g. gym or running.
Transport	Any means of public transportation, such as bus, plane, metro etc.
Wedding	Indoor or outdoor wedding celebration.
Work	In the workplace, typically an office.
Worker dormitories	A shared living space for workers.

The vast majority of these clusters were associated with indoor or indoor/outdoor settings (21/22). Large clusters, such as those linked to churches and ships, were infrequently reported. Almost all clusters involved fewer than 100 cases (181/201), with the outliers being transmission in hospitals, elderly care, worker dormitories, food processing plants, prisons, schools, shopping and ship settings. Religious venues provided a further setting with large cluster sizes: there were separate clusters in South Korea, France, India and Malaysia ( Ananthalakshmi & Sipalan, 2020; BBC, 2020; Salaün, 2020; Shin *et al.*, 2020). In addition to these settings with maximum cluster sizes of more than 100 cases per cluster, we identified five further settings with maximum cluster sizes between 50 and 100: sport (65 cases) ( Korean Centre for Diease Control & Prevention, 2020), bar (80 cases) ( Sim, 2020), wedding (98 cases) ( Ministry of Health – New Zealand, 2020), work (97 cases) ( Park *et al.*, 2020) and conference (89 cases) ( Marcelo & O'brien, 2020).

We found a notably high number of transmission events reported in worker dormitories (21/201), although all of these were from Singapore. This type of setting had the second highest total cluster size out of all the recorded events we found, with 797 cases reported in the S11 dormitory cluster in Singapore ( Data Against COVID19 SG, 2020).

We found only a small number of clusters linked to schools (8/201), and there the SARS-CoV-2 cases reported were most often in teachers or other staff. For example, for two school clusters in Singapore ( Ministry of Health - Singapore, 2020), 16/26 and 7/8 cases were staff. Some children were also found to be infected in these clusters, as was the case in the Salanter Akiba Riverdale school in New York, USA ( Ailworth & Berzon (2020)), although testing for infection was not always universal. In a retrospective close cohort study in a French high school however, 133 children and staff were seropositive for anti-SARS-CoV-2 antibodies, 92 of whom were pupils ( Fontanet *et al.*, 2020).

We identified 9 clusters linked to food processing plants in 4 different countries (USA, Germany, Canada, Netherlands). These transmission events have led to large clusters, such as in a meat processing plant in South Dakota where a total of 518 employees were infected by SARS-CoV-2 ( Cannon, 2020).

The setting with the greatest number of reported clusters of SARS-CoV-2 transmission was households (36/201). Again, most were from China (25/36) with all cluster sizes being less than 10. However, for 27 out of 36 studies, we were unable to calculate either the secondary or final attack rates due to a lack of information on total household size.

We aimed to estimate secondary and final attack rates in other settings but, as for households, we found that there was substantial missing data. In particular, the number of individuals in a setting was missing, and so we were unable to perform this analysis. Where attack rates could be estimated for individual clusters, these are reported in the online database.

Although information on the index and early cases in a setting was often reported, further information on the subsequently reported 10–100 cases in a country was difficult to extract. Moreover, the index cases were often quarantined and hence not linked to further transmission in most settings.

## Discussion

In this review of SARS-CoV-2 transmission events, we found that clusters of cases were reported in many, predominantly indoor, settings. Note that we restrict cluster size to only include individuals infected within a specific setting, and exclude secondary infections which occurred outside the settings. Most clusters involved fewer than 100 cases, with the exceptions being in healthcare (hospitals and elderly care), large religious gatherings, food processing plants, schools, shopping, and large co-habiting settings (worker dormitories, prisons and ships). Other settings with examples of clusters between 50–100 cases in size were weddings, sport, bar, shopping and work. The majority of our reports are from China and Singapore.

### Limitations

The settings collated here are biased due to the nature of our general search for SARS-CoV-2 transmission described above. Although based on a systematic review of published peer-reviewed literature, many of the reports included came from media articles where relevant epidemiological quantities were not always reported, resulting in many missing data. Many of the more detailed studies originated from the early outbreak in China, especially those providing household information. The settings we identified here therefore might not be representative of settings from a global perspective. Bias is present when relying on media coverage - a cluster is more likely to be reported if controversial or if there is an interesting social narrative. This is then compounded by the method search engines use to provide results where priority is given to high traffic stories. Overall, this can lead to some settings being overly represented in our database, which is why the numbers of clusters per settings should be compared cautiously.

Similarly, there is a bias in our reports which means that attendance in settings with many individuals is more likely to be linked to a cluster: recall bias ( Spencer *et al.*, 2017). The accuracy of memories is influenced by subsequent events and experiences such that special, one-off events may be more likely to be remembered and potentially reported. If multiple single transmission events had occurred whilst walking in a park, for example, these would be less likely to be remembered, and more difficult to detect and hence record. Networks of close contacts also tend to be small, resulting in multiple opportunities for transmission, and hence potentially increase the importance of households or workplace for transmission instead of single outstanding settings of potential transmission. Hence, we cannot determine with any reliability the relative importance of the reported different types of settings beyond the record that clusters have been linked to such places.

Other events, such as large music concert ( Dalling, 2020), political ( Jones, 2020) and sporting ( Hope, 2020; Roan, 2020; Wood & Carroll, 2020) gatherings, could potentially have been linked to clusters of COVID-19. But, in the absence of rigorous surveillance systems and widespread testing that would allow countries to link and report the transmissions of such events, such connections remain speculation. An example of this lack of surveillance would be the UK, where only 4/201 clusters have been recorded The outlier for this is Singapore which appears to investigate clusters systematically and provides a well-designed online dashboard with details of all clusters detected ( Data Against COVID19 SG, 2020).

In many settings, only symptomatic cases of disease severe enough to require hospitalization are tested and ultimately reported. This misses those infections that result in mildly symptomatic or asymptomatic symptoms, although there is mounting evidence for a significant proportion of infections to remain asymptomatic ( Gudbjartsson *et al.*, 2020; He *et al.*, 2020; Lavezzo *et al.*, 2020). For some of the clusters, primarily households, all contacts were tested for infection; but for most of the data collated here, the number of COVID-19 symptomatic cases was the only information provided. These reported cases are a subset of all infections and in the absence of more comprehensive data, such as could be collated through widespread cluster investigation and community testing, we cannot conclude anything about clusters of infections, nor that we have included all relevant settings in which transmission can occur. We were also unable to estimate attack rates from the available data, meaning that comparison between rates of transmission in settings is impossible to achieve.

### Settings associated with large cluster sizes

One type of setting that was associated with large numbers of eventual cases was religious venues. The common features of these meetings are the large number of attendees, confined spaces and physical contact. For example, there were eventually more than 5000 COVID-19 cases linked to transmission at the Shincheonji Church of Jesus in South Korea ( Shin *et al.*, 2020). In this particular religious venue, no preventative action was taken despite knowing members were infected with SARS-CoV-2. In other venues, transmission events took place without prior knowledge of any infections and before the WHO declared pandemic status. Other large clusters in this setting type were associated with annual religious events that took place over a few days or weeks ( Ananthalakshmi & Sipalan, 2020; BBC, 2020; Salaün, 2020). Attendees returned to their home countries where they continued to transmit. This generated many secondary cases internationally as well as locally. However, it is clear from smaller “first-generation” clusters, which our analysis focuses on, that these settings provide ideal conditions for transmission: we found 7/16 identified religious clusters had 10 cases or less, whilst 9/16 had 23 or more (see online database https://bit.ly/3ar39ky and *Underlying data* ( Leclerc *et al.*, 2020) for more information). The number of cases in each cluster is an approximation, and little is known about the number of index cases in these religious meetings to begin with, with the exception of the South Korea cluster. Religious events are well known sources of heightened transmission; there is a focus on vaccination recommendations for attendees to the annual Hajj pilgrimage for example, which is currently being postponed for 2020 ( Aljazeera, 2020).

Worker dormitories have been recognised as key places linked to transmission in Singapore, with 893 out of 942 new cases recorded on April 18th being residents in such dormitories ( Asia, 2020). We found 21 reported clusters, one of which had the second largest cluster size of all the events we report here; 797 cases which from the data we believe is a first-generation cluster. Worker dormitories are similar to households ( Dalling, 2020) in the sense that they are places where people live together and come in frequent close contact; however, the number of residents in dormitories is higher than in most other households. This probably contributes to the higher cluster sizes seen in this setting. Additionally, hygiene facilities can be limited in worker dormitories ( Paul *et al.*, 2020), which could also explain the higher transmission. These points also apply to prisons, another type of large co-habiting setting for which we have identified 4 clusters with a maximum cluster size of 353 cases. It would be beneficial to compare attack rates across households, worker dormitories and prisons, to better understand which factors influence the risk of transmission between people who share a living space. Unfortunately, we were unable to identify the total number of residents in these dormitories and prisons, which prevented us from deriving attack rates and making this comparison.

In addition to religious events and worker homes, we also identified clusters of more than 100 cases in elderly care homes, hospitals and ships. These are all known to be at risk of clusters of infectious disease ( Blanco *et al.*, 2019; Kak, 2015; Lansbury *et al.*, 2017). Moreover, people in these settings are often older than the general population and hence at greater risk of severe forms of COVID-19 disease ( U.S Centers for Disease Control and Prevention, 2020). The increased mortality and likely dependence on availability of personal protective equipment (PPE) mean that healthcare clusters are more politically sensitive and hence more likely to be reported.

A more unexpected setting type is perhaps food processing plants, in which we identified clusters of up to 518 cases ( Cannon, 2020). These plants have been the source of clusters in multiple countries. It is possible that the cold atmosphere in this setting has facilitated the spread of the virus ( Molteni, 2020). Other possible explanations include the close proximity of workers for prolonged periods shared welfare spaces, as well as the need to speak loudly to communicate over the noise of the machines, which could lead to an increased projection of viral particles. Another explanation is that we may not be seeing clusters from other manufacturing settings with similar working environments, as fewer have been in operation due to lockdown guidelines during the pandemic, whereas food production has continued.

We identified seven additional setting types with cluster sizes above 50 or 100 cases (school, sport, bar, shopping, wedding, work and conference), which shared characteristics with the settings described above (see online database for more information https://bit.ly/3ar39ky and *Underlying data* ( Leclerc *et al.*, 2020)). Notably, sport, bars, shopping areas and conferences are predominantly indoor settings, where people are in close proximity. For conferences and work, like religious events, transmission within the cluster is facilitated by the duration of the events over several days, as well as the combination of interactions there (workshops, dinners etc…). This can also apply to weddings, where transmission is further increased due to the close-proximity interactions between people (kissing, hugging, dancing etc…). As for bars and shopping areas, these are places with important fluxes of people, which increases the diversity of contacts. Finally, schools, like religious groups, can sometimes represent tightly knit communities which facilitates disease transmission amongst individuals, as was the case with the Salanter Akiba Riverdale school in New York, with a cluster size of at least 60 cases ( Ailworth & Berzon (2020)).

### The first 100 transmission events & under reporting

The pursuit of the first 100 transmission events revealed little on settings of transmission. This reflects the wider issue we found of under reporting and is likely to reflect the fact that many public health surveillance systems were quickly overwhelmed and could not continue outbreak investigations. An example of this is the UK where only limited information on case follow-up and cluster investigation appears to be available. The impact of such under reporting is that we cannot say with certainty what contribution each setting had to overall transmission – we do not have the denominator information on time and contact in all settings. Nor do we have universal screening for detection of all infections, many of which will be asymptomatic. The importance of such universal testing for infection in interpreting whether transmission has occurred in a setting is highlighted by the difference between the low number of clusters linked to schools and the high level of infection reported in one French high school study ( Fontanet *et al.*, 2020).

Further work could pursue data from early investigation of cases where available, to explore the relative importance of different settings to transmission. Importantly, this may counter a bias towards small cluster sizes: with a lack of follow-up only some of the cases actually linked to a setting may be reported and linked. Detailed outbreak investigations should also be explored to get information on the places where transmission is unlikely to have occurred, e.g. if a COVID-19 patient reports 30 contacts at place “A”, “B” and “C”, but only contacts in “C” subsequently become infected this reflects reduced risk in settings “A” and “B”.

### Implications for further work

We found that many clusters of cases were linked to indoor settings, but this may be because early spread in China was during their winter, with people naturally spending more time inside close spaces. Increasing evidence suggests that transmission of SARS-CoV-2 can occur via airborne droplets ( Morawska & Cao, 2020); however, it is likely that outdoor transmission risk is lower ( Nishiura *et al.*, 2020). Further work is needed to clarify this. We found only few clusters in school settings. However, there were many clusters associated with household transmission, and children could be the entry point for the virus into this setting. Although it should be noted in this context that the Report of the WHO-China Joint Mission on Coronavirus Disease 2019 (COVID-19) did not find a single instance where people recalled transmission from a child to an adult ( WHO-China Joint Mission Members, 2020). More generally, the role of children in widespread transmission of the virus is unclear, and whether reopening schools could trigger increased introductions of the virus into households and further within-household spread will have to be carefully monitored.

Further investigation of settings that facilitate clusters of transmission could provide important information for containment strategies as countries lift some of the current restrictions. Previous work has suggested that there might be considerable heterogeneity in individual transmission, which would imply a disproportionate impact from preventing large transmission events from occurring ( Endo *et al.*, 2020). Whilst widespread contact tracing is often considered part of future containment strategies, there is a need for this to be complemented with retrospective investigation of clusters in order to better understand the extent to which certain settings and behaviours are at particular risk of generating clusters of transmission. This could, in turn, inform contact tracing efforts and might be particularly relevant in the context of contact tracing using mobile phone apps, which has recently been suggested in support of more traditional contact tracing ( Ferretti *et al.*, 2020). For example, past co-location in certain settings could be a trigger for notification of risk from an app instead of, or in addition to, individual contacts.

### Online database of collected reports

The online database (accessible at https://bit.ly/3ar39ky) provides information on all collected reports, references and information on cluster sizes as well as notes about the study. This database will be kept as a static source linked to this report, but with an additional tab for newly reported settings. Readers can submit information in the “Suggested updates” tab and we will aim to update information if evidence for substantial new clusters are found linked to a setting that was not in this study.

## Conclusions

In conclusion, we found evidence of SARS-CoV-2 transmission in many types of settings. Our results provide a basis to identify possible places that are linked to clusters of cases and could be closely monitored, for example by linking to app-based contact tracing, and/or remain closed in the first instance following the progressive removal of lockdown restrictions. However, reporting should be improved in the majority of settings, with implementation of systematic reporting on the number of potentially exposed individuals and the number of confirmed and suspected cases from these settings, to allow the estimation of attack rates.

## Data availability

### Underlying data

Figshare: COVID19 settings of transmission - collected reports database. https://doi.org/10.6084/m9.figshare.12173343.v3 ( Leclerc *et al.*, 2020).

This project contains ‘COVID-19 settings of transmission - database.xlsx’, which contains the data extracted from the initial search, as well as an updated version of the dataset from 26/05/2020.

Up to date information on all collected reports is provided in an open-access online database (accessible at https://bit.ly/3ar39ky).

This database provides references and information on cluster sizes as well as notes about the studies.

Data are available under the terms of the Creative Commons Zero "No rights reserved" data waiver (CC0 1.0 Public domain dedication).
