# Supplementary material for: What settings have been linked to SARS-CoV-2 transmission clusters?
Source: Wellcome Open Res. 2020 Jun 5;5:83. Originally published 2020 May 1. [Version 2] doi: 10.12688/wellcomeopenres.15889.2 (PMC7327724; doi:10.12688/wellcomeopenres.15889.2)
Supplement: Peer review history

# Peer review: Reviewer response for version 2

**DOI:** 10.21956/wellcomeopenres.17583.r39003

**Published:** 2020-06-30

**Authors:** Samuel V. Scarpino

**Affiliations:** 1 Network Science Institute, Northeastern University, Boston, MA, USA

In this manuscript, the authors conduct a thorough literature review and identified SARS-CoV-2 transmission clusters.  After assembling their data set, the authors discuss the possible similarities in settings associated with transmission.  As stated, understanding how transmission risk varies across settings is critical for the safe relaxation of measures implemented to control the spread of COVID-19.  This paper provides a valuable resource and synthesis of what is currently known.  I should note that this article has already been evaluated and I believe the authors have adequately addressed the points raised by the previous reviewer.  However, I do have a few additional comments/questions, which I hope the authors find constructive.

1. While Google Sheets is a convenient tool for entering and sharing small data sets, it is not "permanent" and also has the potential to be corrupted or heavily modified.  There is also no easy way for authors to cite the "version" of the sheet used.  The authors do provide a Figshare, but that appears to date back prior to the revised version.  I would strongly suggest regularly archiving a version of the data set and assigning each update a version number.  At a minimum, please provide a DOI for the revised data set.
2. I am concerned that one reason we don't see more evidence for transmission at schools is that schools were closed early in nearly all locations.  To my knowledge, Sweden is not reporting data on whether there have been significant transmission in their schools (as the authors know not all of which are open).  I believe the authors should provide a strong disclaimer, either in the abstract or early in the discussion that we really don't have much to go on w.r.t. schools.  (Of course this is my opinion and likely subject to debate).
3. The authors state, "More generally, the role of children in widespread transmission of the virus is unclear, and whether reopening schools could trigger increased introductions of the virus into households and further within-household spread will have to be carefully monitored."  But, I also feel that given the uncertainty in whether children are import for ongoing transmission, there are other settings we should caveat.
4. The authors note that they, "use peer reviewed articles as a quality threshold," and, while I strongly disagree with the exclusion of pre-prints, I think the authors should at least provide some information on how many studies or clusters were excluded.  Given the long (and increasing lag) between pre-print and publication, is this study missing half of all clusters that are currently published or in-review?  10% 95%?  Providing information around what's been excluded is standard practice for such reviews and feels critical in this case.

Is the work clearly and accurately presented and does it cite the current literature?

Yes

If applicable, is the statistical analysis and its interpretation appropriate?

Not applicable

Are all the source data underlying the results available to ensure full reproducibility?

Partly

Is the study design appropriate and is the work technically sound?

Partly

Are the conclusions drawn adequately supported by the results?

Yes

Are sufficient details of methods and analysis provided to allow replication by others?

Partly

Reviewer Expertise:

Epidemiology

I confirm that I have read this submission and believe that I have an appropriate level of expertise to confirm that it is of an acceptable scientific standard.

# Peer review: Reviewer response for version 2

**DOI:** 10.21956/wellcomeopenres.17583.r38985

**Published:** 2020-06-05

**Authors:** Joël Mossong

**Affiliations:** 1 Epidemiology and Microbial Genomics, Laboratoire National de Santé, Dudelange, Luxembourg

My comments and suggestions have been adequately addressed.

Is the work clearly and accurately presented and does it cite the current literature?

Yes

If applicable, is the statistical analysis and its interpretation appropriate?

Yes

Are all the source data underlying the results available to ensure full reproducibility?

Yes

Is the study design appropriate and is the work technically sound?

Partly

Are the conclusions drawn adequately supported by the results?

Partly

Are sufficient details of methods and analysis provided to allow replication by others?

Yes

Reviewer Expertise:

Epidemiology of infectious diseases

I confirm that I have read this submission and believe that I have an appropriate level of expertise to confirm that it is of an acceptable scientific standard.

# Peer review: Reviewer response for version 1

**DOI:** 10.21956/wellcomeopenres.17429.r38734

**Published:** 2020-05-18

**Authors:** Joël Mossong

**Affiliations:** 1 Epidemiology and Microbial Genomics, Laboratoire National de Santé, Dudelange, Luxembourg

This manuscript aims to provide a descriptive analysis of transmission settings of Covid19 based on published articles or media reports, which is of major interest for controlling the epidemic.

 I have several major concerns: 

1. Most settings reported herein are not representative of settings from a global perspective, most are from the initial epidemic in Asia (mainly from the Singapore dashboard and <20% of settings in the manuscript are outside of Asia). This needs to be added to the discussion as a major limitation.
2. Some important and widely reported outbreaks in particular settings are missing. e.g. the outbreak of the megachurch in Mulhouse France (https://www.dailymail.co.uk/news/article-8168819/French-megachurch-meeting-blamed-sparking-countrys-biggest-cluster-Covid-19-cases.html) and the Ruby Princess outbreak (reported in https://www1.health.gov.au/internet/main/publishing.nsf/Content/1D03BCB527F40C8BCA258503000302EB/$File/covid_19_australia_epidemiology_report_9_reporting_week_ending_23_59_aedt_29_march_2020.pdf ^1^) or the cluster in the french ski resort (https://www.bbc.com/news/uk-51425702). This somehow questions the completeness of the systematic review. The authors could have widened their search terms to include the settings (church, ship, etc.) and outbreak when searching media reports.
3. Given that this manuscript from a team in the UK, it is surprising that only 4 outbreak settings were reported for the UK. The authors need to discuss why they were not able to find more reports from the local and national media outlets in English speaking countries like UK, Ireland, and possibly also Australia, Canada and the US.
4. The authors should discuss reasons for under reporting: public health surveillance systems in many countries were quickly overwhelmed to investigate transmission settings and chains of transmissions. Transmission clusters in elderly care and hospitals homes due to political sensitivity, linked to increased mortality, lack of adequate PPE equipment
5. Meat factories and slaughter houses have recently emerged as high risk setting in the US (https://edition.cnn.com/2020/04/08/business/meat-plant-closures-coronavirus/index.html) and Germany (https://www.dw.com/en/coronavirus-breaks-out-in-third-german-slaughterhouse/a-53389860). This setting should be included separately in Table 1.

Minor comments:

1. Add the sum of cases for all clusters per setting in table 1.
2. p.3.& p. 7 "the first 100 transmission events". While this is an interesting concept, it isn't really being addressed in this article. No country presented herein has collected more than 100 events. The paragraph in the discussion on this seems therefore irrelevant and could be deleted.
3. p. 7. The authors mention that there is increasing evidence for airborne transmission. The current consensus is that most transmission occurs via airborne droplets, which is different to aerosol transmission. I suggest to replace "be airborne" by "occur via airborne droplets".

Is the work clearly and accurately presented and does it cite the current literature?

Yes

If applicable, is the statistical analysis and its interpretation appropriate?

Yes

Are all the source data underlying the results available to ensure full reproducibility?

Yes

Is the study design appropriate and is the work technically sound?

Partly

Are the conclusions drawn adequately supported by the results?

Partly

Are sufficient details of methods and analysis provided to allow replication by others?

Yes

Reviewer Expertise:

Epidemiology of infectious diseases

I confirm that I have read this submission and believe that I have an appropriate level of expertise to confirm that it is of an acceptable scientific standard, however I have significant reservations, as outlined above.

## References

[1] COVID-19, Australia: Epidemiology Report 9: Reporting week ending 23:59 AEDT 29 March 2020. *COVID-19 National Incident Room Surveillance Team*.2020; 10.33321/cdi.2020.44.29 10.33321/cdi.2020.44.29 Reference source 32264802

# Response

**Published:** 2020-06-01

**Authors:** Quentin Leclerc

**This manuscript aims to provide a descriptive analysis of transmission settings of Covid19 based on published articles or media reports, which is of major interest for controlling the epidemic.**

 Thank you for taking the time to review our article. Please note that we have now updated our analysis to include an additional 49 transmission events (201 events total) and 4 new settings type (“Food processing plant”, “Prison”, “Transport” and “Wedding”; 22 setting types total). Some of these new elements overlap with your suggestions. Our Discussion section has also been updated to reflect these new results.

**I have several major concerns: **

1. **Most settings reported herein are not representative of settings from a global perspective, most are from the initial epidemic in Asia (mainly from the Singapore dashboard and <20% of settings in the manuscript are outside of Asia). This needs to be added to the discussion as a major limitation.**

Thank you for raising this point. We already mentioned in the Discussion - Limitations section that many studies originated from the early outbreak in China, but have included an additional sentence there to clarify that this could prevent our results from being directly applicable to other countries. That said, please note that in our updated analysis, 98/201 (50%) events are from China and Singapore, compared to 92/152 (60%) in our original analysis, which improves the coverage of our results.

 The added sentence is “The settings we identified here therefore might not be representative of settings from a global perspective.”

1. **Some important and widely reported outbreaks in particular settings are missing. e.g. the outbreak of the megachurch in Mulhouse France (https://www.dailymail.co.uk/news/article-8168819/French-megachurch-meeting-blamed-sparking-countrys-biggest-cluster-Covid-19-cases.html) and the Ruby Princess outbreak (reported in https://www1.health.gov.au/internet/main/publishing.nsf/Content/1D03BCB527F40C8BCA258503000302EB/$File/covid_19_australia_epidemiology_report_9_reporting_week_ending_23_59_aedt_29_march_2020.pdf1) or the cluster in the french ski resort (https://www.bbc.com/news/uk-51425702). This somehow questions the completeness of the systematic review. The authors could have widened their search terms to include the settings (church, ship, etc.) and outbreak when searching media reports.**

Thank you for suggesting these additional clusters; we have now added the Ruby Princess and the French ski resort events.

 Our initial analysis was focused on trying to find distinct *settings* in which transmission had occurred. Hence we were initially trying to prioritise examples of new *settings *linked to clusters rather than gathering all data on all outbreaks linked to all settings. This has changed somewhat with the open source database and we are happy to act as a gathering point for cluster data.

 For the outbreak in Mulhouse, this falls into the category of events that we do not include in our analysis. This because we are interested in understanding transmission only within specific settings; for example, for a cruise ship, the cluster size we report corresponds to the number of people infected on that ship only, not the people that these might have infected after disembarking. If we included people infected by passengers after disembarking, this would not reflect the “cruise ship” setting, as this additional transmission could occur in a variety of other settings (household, meal etc…).

 We had already highlighted this in the Methods – Outline section, but have now repeated that point at the beginning of the Discussion to hopefully make this distinction clearer (“Note that we restrict cluster size to only include individuals infected within a specific setting, and exclude secondary infections which occurred outside the settings.”)

1. **Given that this manuscript from a team in the UK, it is surprising that only 4 outbreak settings were reported for the UK. The authors need to discuss why they were not able to find more reports from the local and national media outlets in English speaking countries like UK, Ireland, and possibly also Australia, Canada and the US.**

Our initial search was at the end of March. At that time, the number of confirmed cases in the UK was around 20,000, compared to more than 200,000 now. Therefore, there was little information at the time on clusters in these countries compared with Asia, which is why we were less likely to find media reports on that topic for the UK. For similar reasons, we had little information for English-speaking countries. In addition, because of the lack of widespread testing in the UK and/or follow-up of cases, information on clusters does not appear to be widely available in the UK.

 As of 26/05/2020, we have now identified 39 transmission events in English-speaking countries (19% of all the transmission events we have identified so far). Therefore, our updated analysis is more geographically balanced.

1. **The authors should discuss reasons for under reporting: public health surveillance systems in many countries were quickly overwhelmed to investigate transmission settings and chains of transmissions. Transmission clusters in elderly care and hospitals homes due to political sensitivity, linked to increased mortality, lack of adequate PPE equipment**

Thank you for this suggestion. In line with your comments on the “first 100 transmission events” we have adapted the paragraph in the discussion to discuss reasons for under reporting.

 We have also added a sentence to the paragraph on healthcare clusters in the discussion to reflect the likely increased reporting of clusters linked to these settings due to political sensitivity. 

1. **Meat factories and slaughter houses have recently emerged as high risk setting in the US (https://edition.cnn.com/2020/04/08/business/meat-plant-closures-coronavirus/index.html) and Germany (https://www.dw.com/en/coronavirus-breaks-out-in-third-german-slaughterhouse/a-53389860). This setting should be included separately in Table 1.**

Thank you for raising this point. Our online database had been updated to reflect this, and we have now added the “Food processing plant” setting type in our analysis, and comment on this in the Results and Discussion sections of our article.

 This also applies to our new “Prison”, “Transport” and “Wedding” setting types.

**Minor comments:**

1. **Add the sum of cases for all clusters per setting in table 1.**

 We have now implemented this suggestion in the revised article.

1. **p.3.& p. 7 "the first 100 transmission events". While this is an interesting concept, it isn't really being addressed in this article. No country presented herein has collected more than 100 events. The paragraph in the discussion on this seems therefore irrelevant and could be deleted.**

We agree it was frustrating not to find this data, which would have been an interesting angle, giving us “denominator” information. In line with the comments above we have adapted this paragraph to link to under reporting.

1. **p. 7. The authors mention that there is increasing evidence for airborne transmission. The current consensus is that most transmission occurs via airborne droplets, which is different to aerosol transmission. I suggest to replace "be airborne" by "occur via airborne droplets".**

Thank you for this suggestion, we have now rephrased this accordingly.
